# Where are the immunoglobulins? A review of non-secretory multiple myeloma

**DOI:** 10.1007/s12308-025-00652-8

**Published:** 2025-08-06

**Authors:** Marcello Pecoraro Toscano, Megan O. Nakashima

**Affiliations:** Department of Pathology and Laboratory Medicine, Cleveland Clinic, Cleveland, OH U.S.A.

**Keywords:** Non-secretory myeloma, Non-producer myeloma, Oligosecretory myeloma, Multiple myeloma, Plasma cell myeloma, Paraprotein, M protein

## Abstract

Multiple myeloma (MM) is a malignant neoplasm of clonal plasma cells, typically associated with the production of a monoclonal protein. In 1–3% of cases, MM presents without measurable monoclonal protein (M protein) in the serum or urine and normal serum-free light chains; these cases are referred to as non-secretory MM (NSMM). This definition has changed over time according to the sensitivity of laboratory methods for detecting paraproteins. NSMM has been previously reported to have a less aggressive presentation and clinical course compared to secretory MM; however, the literature is conflicting. Recent studies have indicated that NSMM may exhibit different responses to therapy and outcomes, emphasizing the need for a tailored approach. This review consolidates the current understanding of NSMM and underscores the importance of advanced diagnostic techniques in improving patient management and outcomes.

## Introduction

Multiple myeloma (MM) is a malignant neoplasm of clonal plasma cells, comprising at least 10% of a bone marrow biopsy cell population or identified as a biopsy-proven plasmacytoma. Myeloma-defining events, often termed CRAB (hyper*c*alcemia, *r*enal dysfunction, *a*nemia, *b*one lesions) criteria and/or biomarkers of malignancy (SLM, ≥ *s*ixty% bone marrow plasma cells, involved uninvolved serum-free *l*ight chain ratio > 100, one or more lesion detected on *M*RI) [1], differentiate smoldering (no-MM-defining features) from overt/symptomatic MM. As of 2022, MM represents approximately 0.9% of all cancer cases, with a mortality rate of 1.2% [2].

Monoclonal (M) proteins (or paraproteins) are immunoglobulin (IG) molecules secreted by the clonal plasma cells. The immunoglobulin composition can include intact immunoglobulins (appropriately assembled heavy and light chains), free light chains (FLC) without an associated heavy chain, or a mixture of intact IG molecules and FLC [3]. The measurement of circulating M protein is essential for diagnosis and monitoring patients with MM [4].

In a minority of cases, the monoclonal component may not be detectable through standardized laboratory testing, complicating diagnosis and disease monitoring of these patients [5]. These cases are termed non-secretory multiple myeloma (NSMM) and account for approximately 1% to 3% of all MM cases [6, 7]. This review explores the definition, biological characteristics, and clinical implications of NSMM.

### Definition

Non-secretory multiple myeloma is currently defined as MM without an M protein detectable in either serum and urine by electrophoresis with immunofixation and normal serum FLC (sFLC) [1, 8, 9]. Cases of secretory MM may lose detectable paraprotein over time and after therapy; these cases are distinguished from de novo NSMM and are outside the scope of this discussion. The incidence of NSMM was historically considered to be as high as 5% of cases [8, 10, 11], although improvements in the sensitivity of detection of M proteins have reduced the number of cases previously considered NSMM, resulting in a current estimation of 1–3% [1]. Complicating the literature is the fact that previous classifications and historical literature on NSMM have included cases of oligosecretory multiple myeloma (OSMM), in which only a small amount of M protein is produced, too small to be detected if using less sensitive methods [12].

Patients with serum M protein < 1 g/dL and urine M protein < 200 mg/24 h are deemed as having “non-measurable disease” and include cases of both OSMM and NSMM [10, 13]. Serum FLC assays are immunoassays that utilize antibodies specific for epitopes which are obscured when the light chain is bound to the heavy chain. The development of sFLC measurement provided a method with higher sensitivity in detecting IG components, and the definition of measurable disease now includes involved FLC level ≥ 10 mg/dL with an abnormal sFLC ratio [5, 14]. The addition of this criterion reduced the percentage of cases considered NSMM and reduced the number of “non-detectable” cases by over 60% [4, 14]. Serum FLC measurement has been incorporated in the assessment of treatment response and relapse for MM [14, 15]. Heaney et al. compared two sFLC assays and found that 97% of patients with urine FLC below the level recommended for measuring response to treatment had measurable sFLC levels [16]. Both assays showed that more than 35% of cases considered NSMM had measurable serum light chains, thus reclassifying these cases as OSMM.

### Pathogenesis

In 1976, Preud’homme et al. hypothesized that non-secretion of M protein could be due to the plasma cells in NSMM synthesizing truncated IG molecule components; these would not be secreted and would be tagged for quick intracellular degradation [17, 18]. The differentiation between a failure to produce IG or an IG component (non-producer) and/or to secrete it (non-secretory or “non-excretory”) MM has been historically recognized [19].

**Fig. 1:**
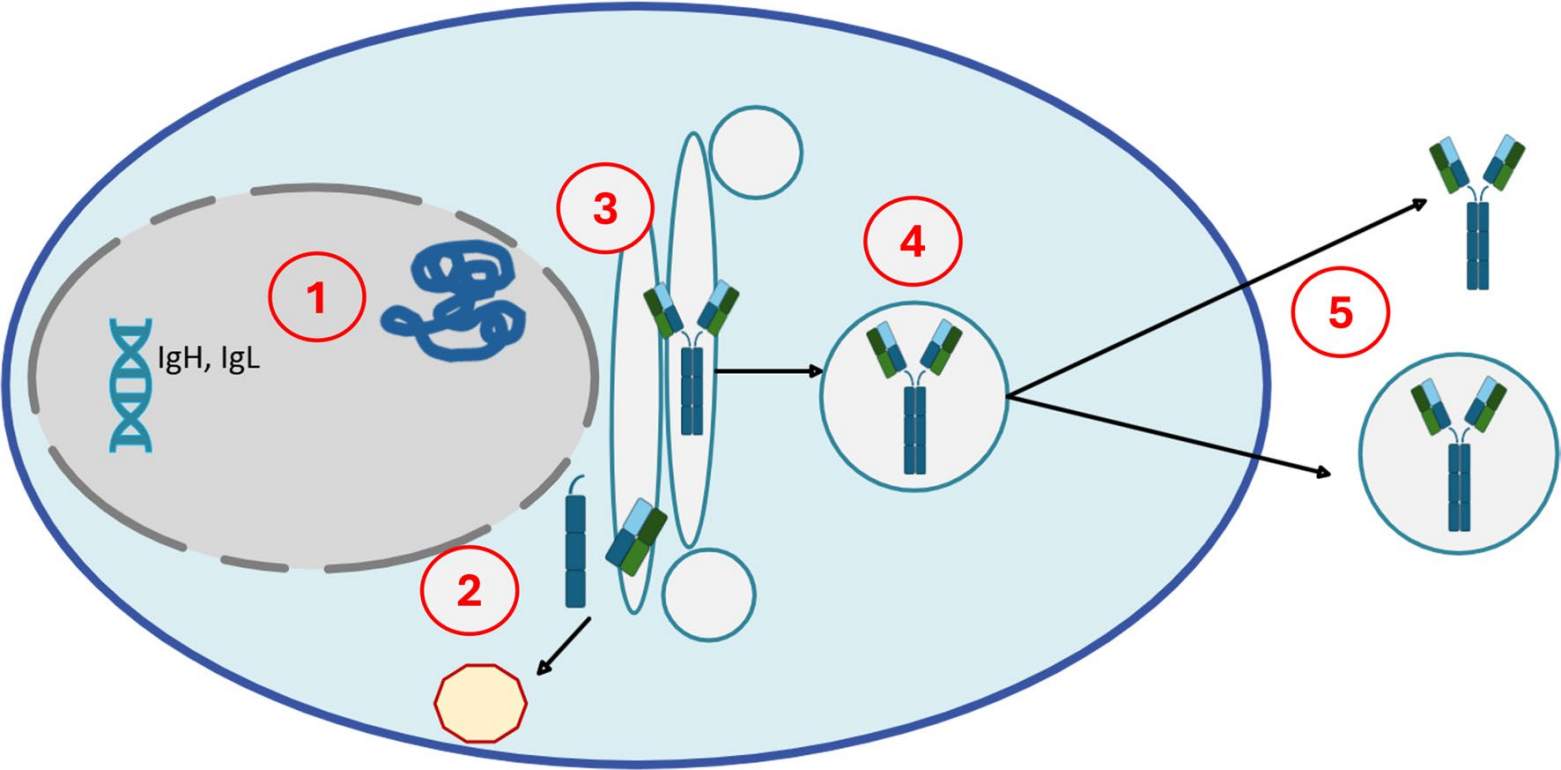
Mechanisms of production and secretion of immunoglobulins and potential errors. (1) mRNA is translated into protein; aberrant transcripts can cause truncated or absent IG components, usually heavy chains; (2) protein folding occurs; errors here could result in a misfolded IG, which is tagged for destruction, often in the proteasome complex; (3) post-translational modifications normally assist in the transport of the assembled IG to the cell membrane; if absent, the vesicle may not reach the cell surface; (4) assembled IG is transported to the membrane surface inside vesicles; (5) correctly assembled IG is secreted from cells, whereas defective IG might persist inside vesicles, undetectable by standard laboratory methods.[3, 20]

We now consider several potential mechanisms which may prevent immunoglobulin production or secretion. Aberrant VDJ rearrangements, splice site mutations, and stop codons may occur and cause truncation or absence of immunoglobulin components, usually heavy chains. Truncated or aberrant IG components may be misfolded and targeted for destruction within proteasomes. There may be errors in post-translational modifications or other processes related to the movement of the protein to the cell surface within secretory vesicles, causing the IG to be “trapped” within the cell or within secretory vesicles. Defective IG molecules might also be undetectable by standard laboratory methods [3, 20, 21] (see Fig. 1).

Structural deviation of IG or IG components from normal has first been described by Coriu et al. who reported a mutation on the gene encoding the constant region of the light chain molecule [22]. This led to its being tagged to and degraded by the proteasome complex. Theories for the absence of secretion include truncation at the LC constant region required for disulfide bonds in the light chain [22] and variable region [23], the result of which is the non-assemblage and/or non-secretion of the immunoglobulin. NSMM also frequently harbors *CCND1*::*IGH* translocations, which lead to a lymphoplasmacytic phenotype and a diminished capacity to secrete [24]. Any aberrant IG secreted within vesicles may still deposit in tissues and cause damage while being undetectable in the serum [25].

In most cases of NSMM, monotypic IG can be detected within the cell by immunohistochemistry or flow cytometry, since at least a portion of the protein is present [26]. If the immunoglobulin protein is not produced, monotypic mRNA should be detectable by in situ hybridization. Aberrant antigen expression (cyclin D1, CD56, CD117) or loss (CD19, CD27, CD81) can also be used to detect the neoplastic plasma cells. Lonial and Kaufman noted that marrow involvement may be the only true objective measure of disease burden, but caution about the potential for patchy disease distribution and possibly non-representative sampling [9].

**Fig. 2:**
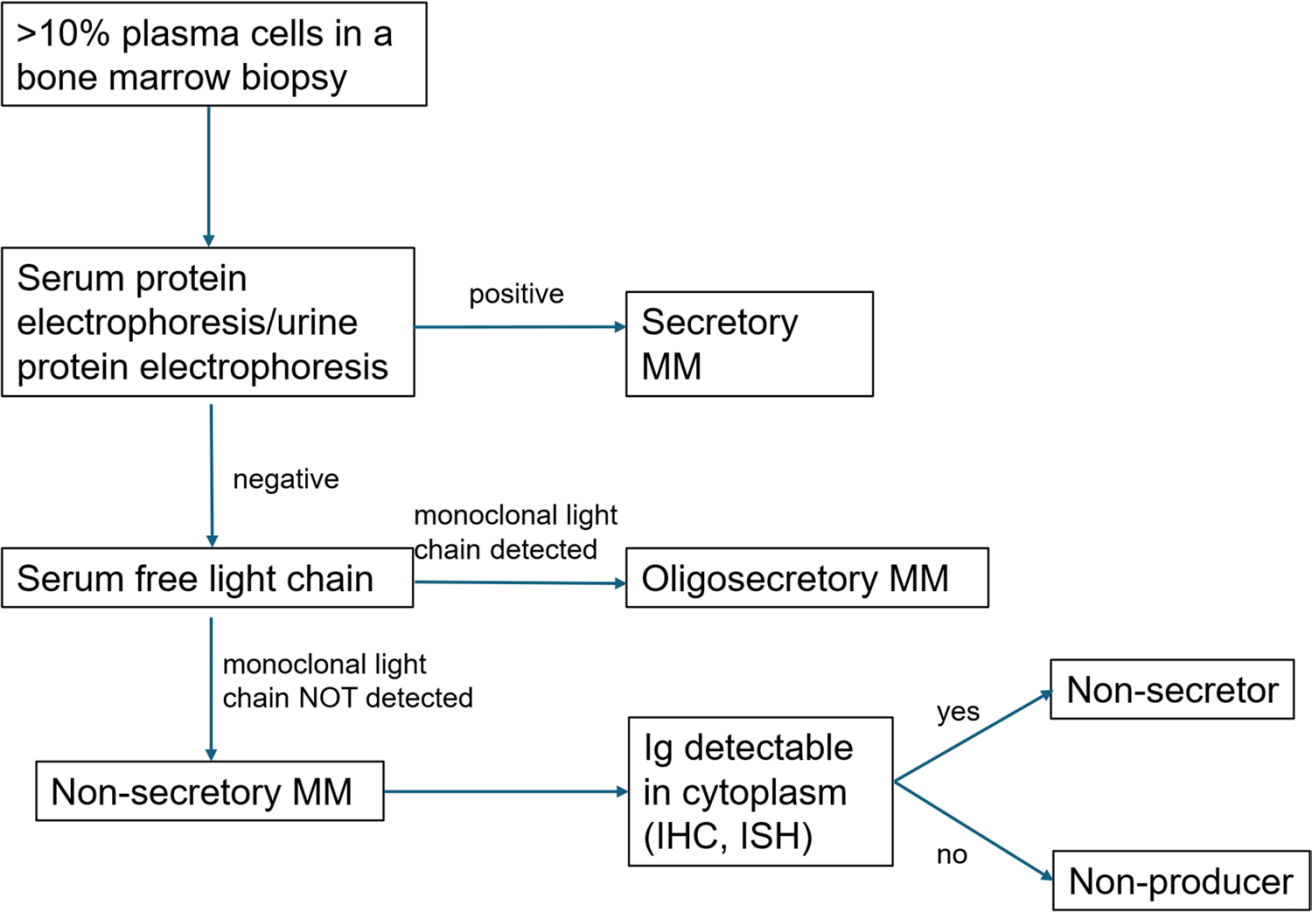
Classification of multiple myeloma according to the secretion of immunoglobulin components

Dupuis and Tuchman proposed the following method for classifying NSMM (see Fig. 2):

- Oligosecretors or light chain-only myelomas
- Non-producers: true absence of any IG production
- Non-secretors: IG molecules produced but unable to be secreted, or secreted within vesicles, preventing detection of the IG in serum

## Implications for disease monitoring and prognostic impact

Since NSMM by definition does not have detectable IG in serum or urine, monitoring is more dependent on PET/CT and bone marrow sampling, rather than the biochemical markers [3]. Bone marrow examination, multiparametric flow cytometry for minimal residual disease, and imaging for tracking skeletal lesions are the best methods to assess response to therapy and minimal residual disease [9].

It has been proposed that NSMM may have a different prognosis compared to secretory MM, but the literature is inconclusive (see summary in Table 1.). This is complicated by the fact that methods for detecting paraproteins have changed over time, making it difficult to compare the results of different studies. Theoretically, NSMM could have a better prognosis due to the absence of light chain deposition in the kidney and other organs and the lack of immunoparesis. Conversely, the absence of detectable paraprotein may also delay diagnosis, leading to a worse prognosis. There may also be other differences in disease biology and response to therapy inherent in NSMM vs secretory MM such as the greater immunogenicity of monoclonal light chain antigens, which elicited a strong type 1 major histocompatibility complex response with T-cell mediated tumor rejection in murine models [3, 26

**Table 1:** Comparison of overall survival (OS) between NSMM and secretory multiple myeloma

Author	Year published	# of NSMM cases	OS of NSMM compared to secretory MM	*P* value of OS difference
Smith	1986^a^	13	Median OS: 46 mo v 21 mo	***P* < 0.01**
Terpos	2003^a^	6	Not reported	*P* = 0.1
Kyle	2003^a^	22	Median OS: 38 mo v 33.4 mo	*P* = 0.60
Kumar	2008^b^	110	Median OS: 69 mo v 59 mo	*P* = 0.34
Chawla	2015	124	8.3 yr v 5.4 yr (patients diagnosed 2001–2012)	***P* = 0.03**
Qin	2019^c^	26	Median OS: 22 mo v 51 mo	***P* < 0.001**
Wålinder	2020^c^	136	Median OS: 44.6 v 42.7	Not significant
Migkou	2020^c^	20	4-yr OS 53% vs 59%	*P* = 0.68
Nandakumar	2020^c^	30	Median OS: 59 mo v 92 mo	*P* = 0.257
Sun	2023^c^	176	Median OS: 81 mo v 70 mo	*P* = 0.401

*NSMM* non-secretory multiple myeloma; *OS* overall survival; *MM* multiple myeloma; *mo* months; *yr* years

^a^Study performed prior to the development of serum-free light chain assays

^b^Study included only patients who had undergone stem cell transplant

^c^Study performed after novel therapies instituted (e.g. lenalidomide, bortezomib)

Notably, NSMM patients may be denied access to clinical trials due to the lack of measurable biomarkers which may be required by study protocols.

Smith et al. [27] reported in 1986 a series of 172 consecutively diagnosed MM in Manchester, UK, with 13 NSMM patients. Overall survival (OS) was superior in the NSMM group compared to secretory MM (46 versus 21 months, *P* < 0.01). However, since this study predates the sFLC assay, it is likely the “NSMM” group also included cases of OSMM.

A 2003 study by Kyle et al. of 1027 MM consecutively diagnosed at Mayo Clinic in the years 1985–1998 found no difference in the OS for 29 NSMM patients versus standard MM cases. Of note, in 5 of 29 NSMM patients, an M protein developed on follow-up [28].

Chawla revisited the Mayo data, including all patients diagnosed 01/1973–06/2012. There were 124 cases of NSMM compared to 6953 patients with “typical” MM. As noted in the Kyle study, in cases diagnosed prior to 2001, OS was similar between NSMM and secretory MM patients. However, the authors found different results when they analyzed the cohort diagnosed between 2001 and 2012, during which time novel therapies such as lenalidomide and proteasome inhibitors came into use. In these patients, OS of NSMM was significantly better than that of secretory myeloma: 8.3 years vs 5.4 years (*P* = 0.03), a difference that remained significant after adjusting for ISS stage [6].

A retrospective study conducted in China by Qin et al. on newly diagnosed MM cases treated with either bortezomib-based or thalidomide-based therapy analyzed MM with unmeasurable M protein (categorized as 61 OSMM, 19 NSMM, and 7 non-producer MM cases). Patients with NSMM had a lower International Staging System (ISS) disease, while those with OSMM showed a higher prevalence of renal dysfunction as compared to the other groups. Plasma cells in cases categorized as non-producers were noted to predominantly have plasmablastic morphology, and these patients were significantly shorter than the other groups (median OS 2.0 months compared to 22.0 for NSMM, 30.0 for OSMM, and 51.0 in measurable disease). NSMM and OSMM had shorter progression-free survival (PFS) and OS than secretory MM, an association that remained significant in multivariate analysis (PFS hazard ratio 2.190; *P* < 0.001, OS hazard ratio 2.441, *P* < 0.001) [29].

Interestingly, when comparing drugs received, bortezomib versus thalidomide, survival was significantly better in patients treated with bortezomib in secretory MM, but there was no difference in survival based on treatment in the non-measurable disease group. The authors speculate this could be due to non-measurable MM cells having levels of IG too low to activate the unfolded protein response (UPR), which is the target of bortezomib [29].

A study of 852 consecutively diagnosed MM in Athens, Greece, and Tel Aviv, Israel, contained 110 OSMM and 20 NSMM patients. NSMM patients tended to be younger, have less anemia, and have lower ISS compared to secretory, but similar 4-year OS. OSMM showed better overall survival (4-year OS of 64% vs 58, *P* = 0.034). However, the OSMM group had lower bone marrow plasma cell infiltration, rates of anemia and hypercalcemia, and ISS stage compared to secretory disease. There was no difference in OS between OSMM and NSMM compared to secretory MM in multivariate analysis adjusting for ISS stage, age, LDH, and high-risk cytogenetics [10].

A study published by Wålinder et al. in 2020 compared survival in 4325 MM patients in the Swedish Myeloma Registry. Secretory MM was compared to non-measurable disease (OSMM and NSMM) [7]. Nine percent of their patients had non-measurable MM, 6% OSMM, and 3% NSMM. There were no statistically significant differences in OS between secretory MM, OSMM, and NSMM. Statistically significant associations included lower ISS stage for OSMM and NSMM, lower percentage of bone marrow plasma cells, and lower creatinine compared to secretory MM. In multivariate analysis, younger age (< 65 years versus ≥ 75 years) was associated with superior survival in OSMM, while low stage and autologous stem cell transplant remained significant for survival in NSMM [7].

Nandakumar et al. reported on Mayo Clinic patients, finding 30 NSMM patients diagnosed from 2008 to 2018. These were compared to a control group of 60 newly diagnosed MM. Contrasting with the other reports, the NSMM patients in this cohort were noted to have a higher tumor burden and higher rate of ISS stage III [30]. Median OS for NSMM was worse than the control group (59 vs 92 months), but this was not statistically significant (*P* = 0.257). The most common genetic abnormality in NSMM was *t*(11;14) (almost 60% of cases), as had been observed by Qin et al. in OSMM and NSMM [29]. The NSMM bearing this translocation had a statistically significant shorter OS (46 months versus 64 months, *P* = 0.003) compared to NSMM cases without this translocation [30].

Sun et al. recently performed a large multicenter retrospective study on NSMM, enrolling 176 NSMM patients in China, and found that the OS difference was not statistically significant between those patients and a control group of secretory MM [31]. Unlike the Nandakumar study, NSMM more often had low ISS stage compared to the control group.

Given the current evidence, the International Myeloma Working Group states that the treatment, response to therapy, and survival rates of NSMM patients are similar to those in secretory MM [8]. Therapy for NSMM is currently the same as for secretory MM.

## Conclusion

Non-secretory multiple myeloma presents a unique challenge in the diagnosis and management of multiple myeloma, accounting for approximately 1% to 3% of MM cases. Advances in diagnostic techniques, particularly the incorporation of serum-free light chain (sFLC) assays, have significantly enhanced the ability to identify patients previously considered to have non-measurable disease, thereby refining this classification. Despite the lack of detectable markers in serum or urine, monotypic IG production can usually be detected in the cells by immunohistochemistry, flow cytometry, or in situ hybridization. Monitoring disease in NSMM includes imaging studies including PET/CT, bone marrow aspiration and biopsy, and assessment of minimal residual disease by techniques such as flow cytometry. NSMM may exhibit a less aggressive course compared to secretory MM, potentially due to the absence of organ dysfunction caused by paraproteins, such as renal insufficiency. However, the prognosis for NSMM remains a topic of debate, with studies showing varying impacts on overall survival and progression-free survival rates. Further studies will better elucidate the clinical significance of NSMM and OSMM and refine treatment strategies, as well as explore biological mechanisms underlying these variants of MM. This could ultimately enhance patient outcomes and facilitate access to clinical trials for those affected by NSMM.

## Data Availability

No datasets were generated or analysed during the current study.
